# 

**Published:** 1856-04

**Authors:** 


### AMERICAN MEDICAL ASSOCIATION.

The ninth annual meeting of the above Association, will be held in Detroit, Michigan, on Tuesday, May 6th, 1856. The right of representation is defined as follows:

1. Local societies to send a delegate for every ten of its members, and one for every fraction over five.

2. The Faculty of every regularly constituted school of medicine, two delegates. A hospital with 100 patients, two delegates.

3. The army of the United States, as well as the staff of the Navy is also provided for.

IOWA STATE MEDICAL SOCIETY.—A meeting of the State Medical Society will be held in Ottumwa, Wapello County, in this State, on the second Wednesday of June next, at 2 o'clock, P. M.

